# Chemical Attraction of Gall Midge Pollinators (Cecidomyiidae: Cecidomyiinae) to *Anthurium acutangulum* (Araceae)

**DOI:** 10.1007/s10886-022-01349-3

**Published:** 2022-03-08

**Authors:** Florian Etl, Wittko Francke, Jürg Schönenberger, Stefan Dötterl

**Affiliations:** 1 Department of Botany and Biodiversity Research, University of Vienna, Vienna, Austria; 2 Institute of Organic Chemistry, University of Hamburg, Hamburg, Germany; 3 Department of Environment & Biodiversity, Paris Lodron University of Salzburg, Salzburg, Austria

**Keywords:** Chemical communication, Cucumber aldehyde, Flower scent, Gall midge Pollination, Spiroacetals

## Abstract

Flowering plants often use chemical signals to attract their pollinators, and compounds that elicit attraction are known for several groups of pollinators. For other pollinators such as gall midges, however, compounds responsible for their attraction to flowers are largely unknown. Here, we describe the pollination biology of *Anthurium acutangulum*, a Neotropical aroid species found to be attractive to gall midges. We collected and analyzed its floral scent by dynamic headspace collections and gas chromatography coupled to mass spectrometry, and identified compounds responsible for pollinator attraction. The inflorescences were almost exclusively visited by gall midges (females; Cecidomyiidae: Cecidomyiinae) and released a strong scent reminiscent of freshly cut cucumber, mainly (5*S*,7*S*)-*trans*-conophthorin, (*E*2*,Z*6)-2,6-nonadienal, and *cis*-conophthorin. Behavioral assays with the two most abundant compounds identified (*E*2*,Z*6)-2,6-nonadienal as being highly attractive to the female gall midge pollinators, whereas (5*S*,7*S*)-*trans*-conophthorin was not attractive. Overall, we introduce a new specialized gall midge pollination system and identify the chemical mediating communication between the pollinators and their host plants.

**Supplementary Information:**

The online version contains supplementary material available at 10.1007/s10886-022-01349-3.

## Introduction

Many vertebrate and non-vertebrate pollinators are attracted to their host plants by, among other cues, floral scents (Raguso 2008). These volatile organic compounds are highly diverse, with more than 2000 components described so far (El-Sayed 2021; Knudsen et al. 2006). Attractive compounds are known for several groups of animal pollinators, such as mammals, bees, and beetles (Dötterl et al. 2012; Raguso 2008), while attractive scents of other taxa, such as gall midges (Cecidomyiidae), have not yet been identified, despite knowing that olfactory cues are often key attractants (e.g., Gardner et al. 2018).

Plants mainly pollinated or co-pollinated by gall midges occur in various lineages, such as Schisandraceae (e.g., *Illicium* spp.), Malvaceae (e.g., *Theobroma cacao*), and Araceae (Young 1985, 1986, Luo et al. 2010; Schwerdtfeger et al. 2002). In Araceae, several species of *Anthurium* are pollinated by these insects (Schwerdtfeger et al. 2002). The plants often attract high numbers of midges, and it was hypothesized that floral scents guided the midges to the inflorescences of these plants. However, floral scents have not been studied for any gall midge pollinated *Anthurium* species so far, and it is currently unclear how these interacting organisms communicate.

During recent observations of *Anthurium acutangulum* Engl. at the “La Gamba Tropical Research Station” in Costa Rica, we observed that inflorescences are frequently visited by gall midges at night. Here, we describe the pollination biology of *A. acutangulum*, and elucidate the chemical communication between this plant species and its gall midge pollinators. Specifically, we asked: 1) What are the main characteristics of the process of anthesis and the pollination biology of *A. acutangulum*? 2) Which floral scent compounds are released by the inflorescences? 3) Are the main compounds attractive to the midges?

## Methods and Materials

### Plant Material and Study Site

**Fig. 1:**
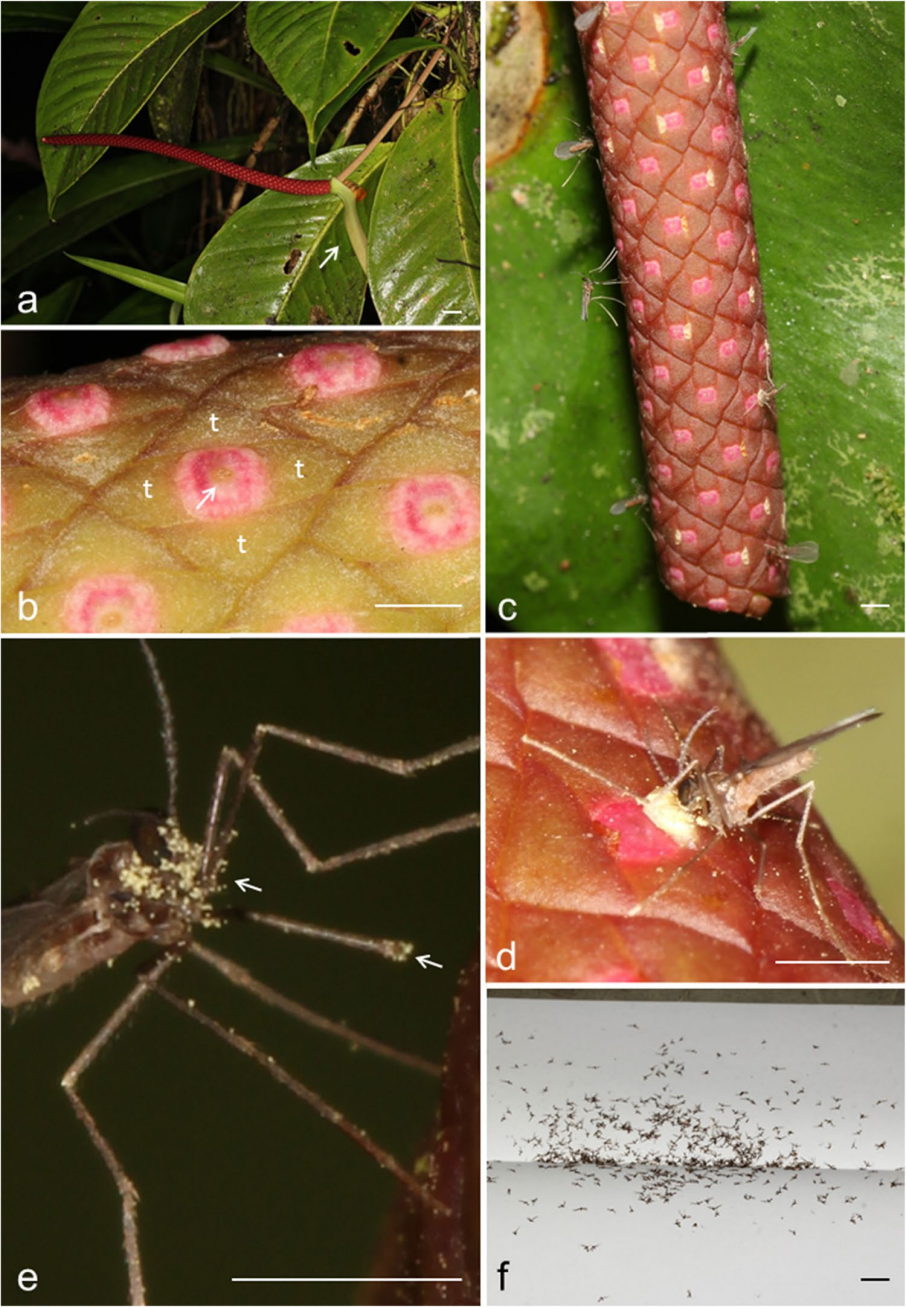
Inflorescence, course of anthesis, and pollinators of *Anthurium acutangulum*. A) Flowering habit of *A. acutangulum* with a purple spadix and a greenish spathe (arrowhead). B) Detail of a female phase inflorescence with tightly packed flowers having moist and receptive stigmas (arrowhead); the stigma is surrounded by pink distal parts of the pistils; stamens are not visible during the female phase and each gynoecium is surrounded by four tepals (t). c, d) Male phase inflorescence with flowers having open anthers, visible by their yellow pollen; several gall midge individuals (Cecidomyiidae: Cecidomyiinae) are feeding on pollen. E) Gall midge with numerous pollen grains of *A. acutangulum* on its head, thorax, and legs (arrowheads). F) Numerous individuals of the gall midge pollinators that were attracted to a filter paper containing synthetic (*E*2,*Z*6)-2,6-nonadienal during three hours of trapping using a BG-Sentinel trap. 1a,f) scale bar = 1cm, 1b-e) scale bar = 1mm.

*Anthurium acutangulum* (Araceae; sect. Porphyrochitonium) occurs from Panama to Honduras in wet tropical forests, from sea level up to 900m asl. It is an epiphyte that frequently grows on the trunks of small understory trees. The flowers are hermaphroditic, protogynous, and arranged in several spirals around a spike, called a spadix (Fig. 1). The study was carried out on five individual plants between February and September of five successive years (2015–2019) at the edge of the Piedras Blancas National Park, in and near the La Gamba Tropical Research Station, Costa Rica (08°42′02′′N, 83°12′07′′W). Vouchers of the plant species are deposited at the herbarium of the University of Vienna, Austria (WU).

### Anthesis, Floral Morphology, Fruiting

Floral morphology during female and male phase and timing of anthesis were studied in all five individuals on one inflorescence each. Therefore, inflorescences were observed daily, starting prior to the beginning of anthesis, in the morning, in the evening, and at night to check for flower opening. Female and male sexual phases were determined by either the presence of moist stigmas or open anthers. Post-anthetic inflorescences were checked two weeks after anthesis for pollination success, i.e. if inflorescences were aborted or if fruits had started to develop (indicated by enlarged carpels).

### Scent Collection and Analyses

Using dynamic headspace methods, we collected scents from four male and one female stage inflorescence (N = 5 individuals) of *A. acutangulum* during night-time (11:00 PM to 3:30 AM), when plants were strongly scented to the human nose and attracted high numbers of gall midges. Inflorescences were bagged with a polyethylene oven bag (10 × 30 cm; Toppits, Germany) and the scent was trapped for 2-3 min directly after bagging on an adsorbent tube (quartz glass tube: length 25 mm; inner diameter 2 mm) filled with 1.5 mg each of Carbotrap B (mesh 20–40, Supelco, Germany) and Tenax TA (mesh 60–80; Supelco, Germany). For scent collection, a membrane pump (Gardner Denver, Germany) was used, and the flow was set at 200 ml/min. To obtain negative controls, we conducted the same procedure, but with empty oven bags (N = 5; see also Etl et al. 2016). Samples were stored in a freezer (-20 °C) and were analyzed ca. two weeks later with a GC/MS (coupled gas chromatograph/mass spectrometer; QP2010Ultra, Shimadzu Corporation, Japan) coupled to a thermal desorption unit (TD-20, Shimadzu, Japan) and equipped with a ZB-5 fused silica column (5 % phenyl polydimethylsiloxane; 60 m long, inner diameter 0.25 mm, film thickness 0.25 μm, Phenomenex, USA). Samples were desorbed at 250 °C for 15 min (flow: 25 ml/min) and cryo-focused on a cold trap at -20 °C in the TD-20, before they were transferred to the GC (cold trap heated to 250 °C, transfer line from TD-20 to GC set to 260 °C). Samples were run at a column flow (carrier gas: helium) of 1.5 ml/min. GC oven temperature started at 40 °C, then increased by 6 °C per min to 250 °C, and was held for 1 min. The MS interface was set at 260 °C, and the ion source at 200 °C. Mass spectra were taken at 70 eV (in EI mode) from *m/z* 30 to 350. The GC/MS data were processed using GCMSolution Version 4.11 software (Shimadzu Corporation, Japan). Compounds were tentatively identified by matches with the NIST 11, Wiley 9, FFNSC 2, Essential Oils, and Robert P. Adams 2007 mass spectral data bases, and were confirmed by comparison of mass spectra and retention times with those of authentic standards available in the stock collections of WF and SD. To determine the amount of scent in the samples, known amounts of monoterpenes, aliphatics, and aromatics were injected into the GC/MS system; mean peak areas of these compounds were used to determine the total amount of scent (see Dötterl et al. 2012).

To obtain a sample for enantioselective analyses of *trans*-conophthorin, scent was collected from a male stage inflorescence of *A. acutangulum* for three hours on a larger adsorbent tube, filled with 15 mg each of the two different adsorbent materials. The trapped compounds were eluted with 1 mL acetone (pro analysi, Merck, Germany). Aliquots of 1 μL of this sample, as well as of (5*S*,7*S*)-*trans*-conophthorin and (5*R*,7*R*)-*trans*-conophthorin (both available in the stock collection of WF) were injected, following Brandt et al. (2019), at a temperature of 250 °C into an Agilent 7890A gas chromatograph (Santa Clara, California, USA) equipped with a fused silica capillary column (30 m × 0.23 mm i.d.), coated with a 0.23 µm film of 0.4 % heptakis(2,3-di-*O*-methyl-6-*O*-*tert*-butyldimethylsilyl)-*beta*-cyclodextrin (DIME-*beta*-CD) (30 %) in SE-52 (70 %). The GC oven temperature increased from 40 °C to 200 °C at a rate of 20 °C per minute.

### Observation of Floral Visitors

The same five inflorescences of *A. acutangulum* as used for the analyses described above were observed for 15 min every two-hours for several days during both day and night during the female and male phases. All insects that visited the inflorescences were recorded and a subset of specimens were captured with an insect-aspirator to look for pollen on their bodies and for later identification. Vouchers of the insect species are deposited at the University of Vienna, Austria.

### Bioassays

**Fig. 2:**
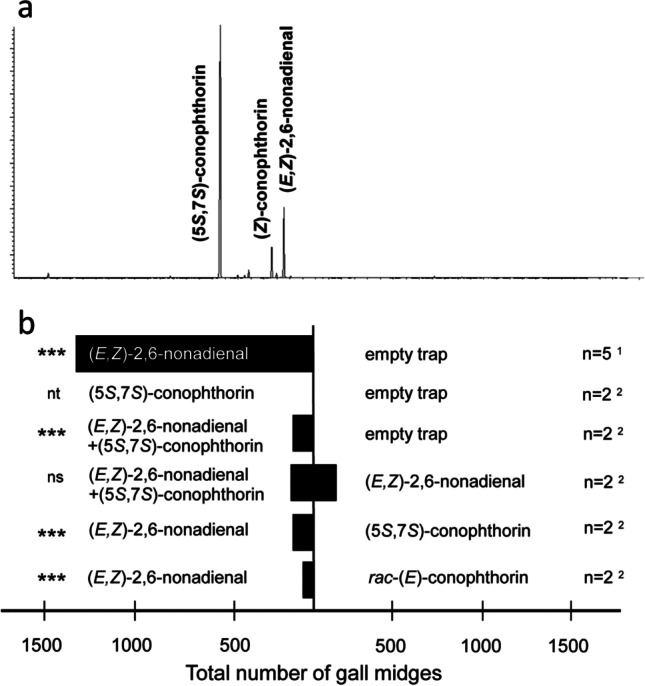
**a** GC/MS chromatogram (total ion current) of a scent sample taken from a male phase inflorescence of *Anthurium* *acutangulum* at night. The sample is dominated by (5*S*,7*S*)-*trans*-conophthorin, (*E*2*,Z*6)-2,6-nonadienal, and *cis*-conophthorin, and contained several other compounds in minor quantities (see Table S1); **b** results of a series of two-choice field bioassays, testing the activity of synthetic (*E*2*,Z*6)-2,6-nonadienal and (5*S*,7*S*)-*trans*-conophthorin alone, each against negative controls, against each other, and against a combination of both compounds as well as against synthetic racemic *trans*-conophthorin. ^1^: All individuals attracted during the choice assay were trapped for 30 min (n=3) or a duration of 3 hr (n=2). ^2^: Values give the mean number of attracted individuals of gall midges at three specific time points during 30 min experiments (see Methods and Materials for more details). Exact binomial tests: ***: *P* ≤ 0.001; ns: not significant; nt: not tested

We tested synthetic (*E*2*,Z*6)-2,6-nonadienal (Sigma Aldrich, USA ≥96%; N=5) and (5*S*,7*S*)-*trans*-conophthorin (N = 2), both main compounds in the scent of *A. acutangulum* (see Results) in two-choice bioassays against negative controls (empty filter paper). (*E*2*,Z*6)-2,6-nonadienal was also tested against (5*S*,7*S*)-*trans*-conophthorin (N = 2) as well as a racemate of *trans*-conophthorin (N = 2). Further, a mixture of (*E*2*,Z*6)-2,6-nonadienal and (5*S*,7*S*)-*trans*-conophthorin was tested against (*E*2*,Z*6)-2,6-nonadienal (N = 2) and against a negative control (N = 2). Pure compounds (200 µL each) were tested in order to obtain emission rates comparable to that of a single inflorescence of the plant (see also Dötterl et al. 2012; Etl et al. 2016). Bioassays were performed within the flowering period of *A. acutangulum* in the study population, started at 9:00 PM, and lasted until 9:30 PM (N = 13) or until midnight (N = 2, see details below). In our experiments, where we tested (*E*2*,Z*6)-2,6-nonadienal against negative controls, arriving insects were either collected with an insect-aspirator and the use of a head torch every 5 min over a period of 30 min (N = 3) or were constantly trapped by BG-Sentinel traps (Biogents, Germany) that were placed 5 cm below the filter papers (N = 2) for a period of 3 hr. As the arriving midges were easily scared away by light and BG-Sentinel traps were not available for further assays, for all other experiments we used a camera, equipped with infrared light (Sony HDR-PJ780), and counted the midges that were gathering on and around (10 - 20 cm) the filter papers every 10 min over a period of 30 min without catching them. With this method, we could not exclude counting the same individuals more than once within the three counts, and so we calculated mean values for the three counts of each of the ten experiments (see also Fig. 2 for more details). The (mean) numbers of insects attracted in the replicates of a specific two-choice assay were pooled for exact binominal tests of goodness-of-fit (http://www.biostathandbook.com/exactbin.xls) that compared the (mean) number of gall midges attracted by different lures.

## Results

### Anthesis and Floral Morphology

Flowering individuals of *A. acutangulum* were found in both the dry (January – March) and the rainy (July, August) seasons. Inflorescences consist of a dark red (Fig. 1a), sometimes more yellowish spadix, which is enclosed by a green spathe during development. The numerous, ca. 3 mm small flowers, which consist of a pistil surrounded by four conically shaped tepals, are tightly packed around the spadix. During anthesis, the spadix reaches a length of 15-20 cm, a diameter of 1.5 cm at its base and 1 cm at its tip (Fig. 1a).

The female phase starts when the yellowish stigmas of almost all flowers synchronically open along the spadix. Receptivity is indicated by the wet appearance of the stigmas (Fig. 1b). Each night, from around 9:00 PM (ca. 3 hr after nightfall), until sunrise (5:00 AM), the spadix continuously emitted a strong scent. This female phase with nocturnal scent production lasted 4-6 days, after which the stigmas wilted.

Around 24 hr after wilting of the stigmas, the male phase started, i.e., the first anthers emerged from the surface of the spadix and released pollen (Fig. 1d). Within the following 12-14 nights, individual flowers started their male phase in an apparently random pattern along the spadix, with only one of the four anthers of a given flower opening per night. During each night a strong scent emission was perceived, similar to that of the female phase.

Two of the five inflorescences observed for fruiting aborted the spadix about two weeks after the end of anthesis. The other three inflorescences developed into infructescences with pink berries that were eaten by males of the orange collared manakin [*Manacus aurantiacus (Salvin, 1870)*].

### Floral Scent

The floral scent of *A. acutangulum* was reminiscent of a freshly cut cucumber. We detected between 0.3 µg and 8 µg of scent and between three and 34 (overall 36) compounds in the samples (Table S1). Most of the compounds occurred only in small amounts, and only three compounds constituted more than 5% to the total scent discharge in at least one sample: (5*S*,7*S*)-*trans*-conophthorin (77%, 62%-98%; mean, min-max), (*E*2*,Z*6)-2,6-nonadienal (10%, 0-24%), and *cis*-conophthorin (7%, 2-13%) (Fig. 2a).

### Floral Visitors

During the female and male phases (in total ca. three weeks), numerous (up to 20 at the same time and a total of on average 33 within an hour) female gall midges of a single morphotype (Cecidomyiidae, Cecidomyiinae) visited the inflorescences at night from ca. 9:00 PM until 5:00 AM (Fig. 1c). The midges landed on the spadix and, while directing their head to the surface of the spadix/flowers (Fig. 1d), crawled along the spadix from flower to flower, feeding on stigmatic exudates of the female phase and on pollen of the male phase inflorescences (Video S1). While feeding on male phase flowers, they became heavily loaded with pollen (Fig. 1e). Each midge spent at least a minute crawling up and down the spadix. The midges were also observed hanging from silky threads of unknown origin nearby the inflorescences, from where they kept flying back and forth to the spadix (Video S1).

Other visitors, which were rare, included one individual of another morphotype of gall midge, several individuals of ants patrolling on the inflorescences, two katydid (bush cricket) nymphs, and a predatory land-flatworm (Plathelminthes: Geoplanidae) that attacked and fed on the gall midge pollinators (Fig. S1).

### Bioassays

In total, more than one thousand female midges of the same morphotype as the ones found on the plants were attracted by (*E*2*,Z*6)-2,6-nonadienal alone (Figs. 1f, 2b), and by a mixture of (*E*2*,Z*6)-2,6-nonadienal + (5*S*,7*S*)-*trans*-conophthorin when each was tested against a negative control (Fig. 2b). No midge responded to the negative controls and no midges were attracted when testing (5*S*,7*S*)-*trans*-conophthorin alone against a negative control. When testing (*E*2*,Z*6)-2,6-nonadienal against (5*S*,7*S*)-*trans*-conophthorin and against *racemic trans*-conophthorin, midges only responded to (*E*2*,Z*6)-2,6-nonadienal. Also, this compound alone attracted approximately the same number of midges as a mixture of (*E*2*,Z*6)-2,6-nonadienal + (5*S*,7*S*)-*trans*-conophthorin (Fig. 2b). No insects other than the female gall midges were attracted during the bioassays.

## Discussion

Our study showed that inflorescences of *Anthurium acutangulum* release a strong cucumber-like scent at night and are highly attractive to female gall midge pollinators at this period of the day. The spiroacetal (5*S*,7*S*)-*trans*-conophthorin and (*E*2*,Z*6)-2,6-nonadienal were overall the most abundant compounds of the floral scent blend, of which (*E*2*,Z*6)-2,6-nonadienal resembled the scent of freshly cut cucumbers. Field bioassays revealed that (*E*2*,Z*6)-2,6-nonadienal attracted high numbers of the gall midge pollinators, whereas (5*S*,7*S*)-*trans*-conophthorin was not attractive.

Based on the fact that the inflorescences of *A. acutangulum* were almost exclusively visited by a specific morphotype of gall midge, which visited female phase inflorescences, touching the receptive stigmas, and getting loaded with pollen while visiting male phase inflorescences (Fig. 1e), we classify these midges as the only effective pollinators of the studied plant population. Thus, we add *A. acutangulum* to the list of *Anthurium* species pollinated by this group of insects (Schwerdtfeger et al. 2002). In contrast to other species in this genus, *A. acutangulum* is visited by gall midges only at night and not during both day and night. Also, we did not observe the midges ovipositing into the flowers, a behavior known from other plants pollinated by gall midges, such as *Artocarpus heterophyllus* (jackfruit, Moraceae; Gardner et al. 2018) and Schisandraceae species (Luo et al. 2010).

The scent released by the inflorescences of *A. acutangulum* is highly specific and we are not aware of another plant species that releases a scent dominated by (5*S*,7*S*)-*trans*-conophthorin (together with *cis*-conophthorin) and (*E*2*,Z*6)-2,6-nonadienal (Knudsen et al. 2006). It strongly differs from the scent released from inflorescences of *Artocarpus heterophyllus*, the only other plant where gall midges have been shown to respond to olfactory floral cues (Gardner et al. 2018). The scent of this Moraceae species is dominated by methyl 2-methylbutyrate, methyl isovalerate, and methyl tiglate, compounds that are not released by *A. acutangulum*. It obviously also differs from other *Anthurium* species that are pollinated by gall midges because these species are described as having flowers that are scentless to the human nose (Schwerdtfeger et al. 2002). Interestingly, however, a cucumber-like floral scent is emitted by *Liparis viridiflora* (Kaiser 1993). Flowers of this orchid release mainly (*E*,*E*)-α-farnesene, a compound not detected in samples of *A. acutangulum*, but also conophthorin and (*E*2*,Z*6)-2,6-nonadienal. Species of *Liparis* are pollinated by small Diptera, among them potentially gall midges. The shared occurrence of these compounds in *A. acutangulum* and *L. viridiflora* might suggest convergent evolution in response to gall midge pollination. This, however, would imply that both compounds elicit responses from gall midges. However, in our study only (*E*2*,Z*6)-2,6-nonadienal attracted the gall midge pollinators, and it remains to be tested whether conophthorin is attractive to other gall midge species at other locations. That is, the large amount of floral scent released by *A. acutangulum* might be explained if it is competing with other sympatric species that also attract the same pollinator species at other sites.

To conclude, we have described a new specialized and mutualistic gall midge pollination system with pollen and stigmatic exudates as floral rewards (see also Yukawa et al. 2011). Also, we provide evidence that (*E*2*,Z*6)-2,6-nonadienal, a compound otherwise rare among floral scents (Knudsen et al. 2006) and not previously known to be involved in the chemical ecology of gall midges (Francke 2013; Hall et al. 2012), was highly efficient in attracting its female gall midge pollinators. It awaits determination in future studies whether this chemical is the only floral scent compound of *A. acutangulum* involved in the attraction of its pollinators, or if other compounds such as *cis*-conophthorin might be active as well. Also, it would be interesting to know whether (5*S*,7*S*)-*trans*-conophthorin, the most abundant compound in the floral scent blend of *A. acutangulum*, has a function other than attraction of pollinators from distance, such as eliciting feeding behaviors in the gall midges, or repelling other potential flower visitors (e.g., florivores).

## Supplementary Information

Below is the link to the electronic supplementary material.

Supplementary file1 (DOCX 180 KB)

Supplementary file2 (ZIP 61319 KB)

Supplementary file3 (XLSX 13 KB)

## Data Availability

All data are included in the manuscript
