# Supplementary material for: Chemical Attraction of Gall Midge Pollinators (Cecidomyiidae: Cecidomyiinae) to Anthurium acutangulum (Araceae)
Source: J Chem Ecol. 2022 Mar 8;48(3):263–9. doi: 10.1007/s10886-022-01349-3 (PMC8934759; doi:10.1007/s10886-022-01349-3)
Supplement: Supplementary file 1 — Supplementary file1 (DOCX 180 KB) [file 10886_2022_1349_MOESM1_ESM.docx]

**Supplementary Figures**


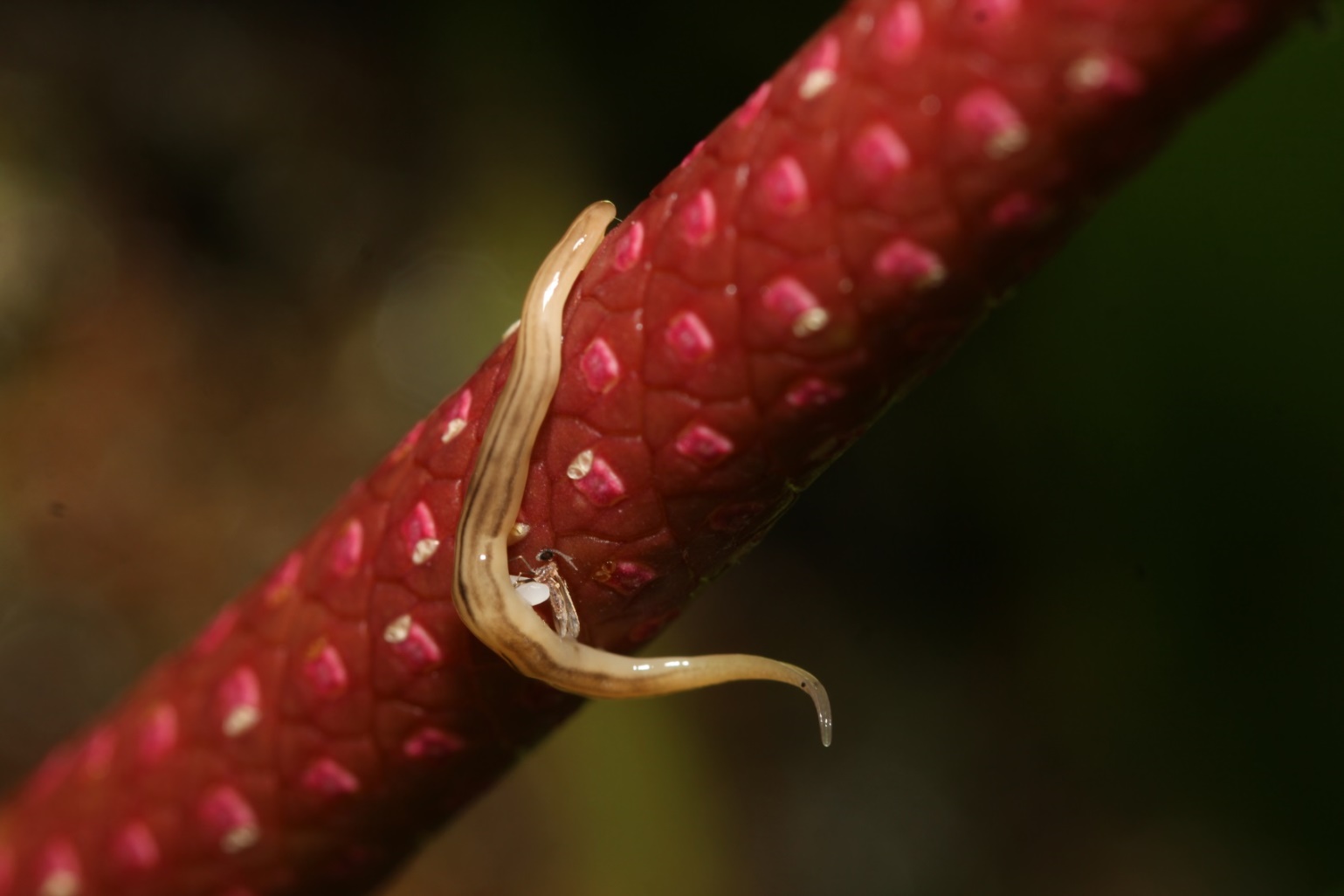


**Fig. S1** A land-flatworm (Plathelminthes: Geoplanidae) feeding on a gall midge pollinator.
